# High bacterial load of indoor air in hospital wards: the case of University of Gondar teaching hospital, Northwest Ethiopia

**DOI:** 10.1186/s40248-016-0061-4

**Published:** 2016-07-05

**Authors:** Zemichael Gizaw, Mulat Gebrehiwot, Chalachew Yenew

**Affiliations:** Department of Environmental and Occupational Health and Safety, Institute of Public Health, College of Medicine and Health Sciences, University of Gondar, Gondar, Ethiopia

**Keywords:** Indoor air, Bacterial load, Hospital wards, Settling plate, Mannitol test, Bacitracin test

## Abstract

**Background:**

The air inhaled by people is abundantly populated with microorganisms which also are called bioaerosols. Bioaerosols is a colloidal suspension, formed by liquid droplets and particles of solid matter in the air, whose components contain or have attached to them viruses, fungal spores and conidia, bacterial endospores, plant pollen and fragments of plant tissues. They account for 5–34 % of indoor air pollution.

**Methods:**

A cross-sectional study was conducted to assess the bacteriological concentration and to identify specific species of bacteria in the indoor air of Gondar University teaching hospital. Air samples were taken from 14 randomly selected wards. Bacterial measurements were made by passive air sampling technique i.e., the settle plate method. In each ward five Petri dishes were exposed for 30 and 60 min in the morning and afternoon. Bacteria were collected on nutrient agar and blood agar media. Both quantitative and qualitative analyses were conducted. The quantitative analysis was mainly conducted to determine bacterial load or number of bacteria in the indoor air. Bacterial load was enumerated as colony forming units. Qualitative analysis was conducted to identify specific species of bacteria. For this study we have selected *Staphylococcus aureus and Streptococcus* which had high public health concern. Mannitol test was used to isolate *Staphylococcus aureus*, whereas Bacitracin test was conducted to isolate *Streptococcus pyogene*.

**Result:**

The result of this study indicated that the highest bacterial load which was 1468 CFU/m^3^ has been recorded at 2:00 PM in Ward C at 60 min exposure time and the lowest bacterial concentration (i.e., 480 CFU/m^3^) was recorded at 8:00 AM in physiotherapy ward. Based on the result bacterial concentration of indoor air of Gondar University teaching hospital was found between 480 and 1468 CFU/m^3^. The result of one way ANOVA showed that the highest mean bacterial concentration (1271.00 CFU/m^3^) was found in Medical ward and the least (583.25 CFU/m^3^) concentration was found in ward D and the grand total average concentration was 878.43 CFU/m^3^. Favorable conditions for growth and multiplication of bacteria like temperature (26.5–29.5 °C), humidity (64.5–85 %), presence of unhygienic attached toilets, poor waste management system and poor ventilation system were observed during the survey. *Staphylococcus aureus* was identified in 10 wards and *Streptococcus pyogenes* was isolated in 8 hospital wards.

**Conclusions:**

Compared with different indoor air biological standards, higher concentration of indoor air bacterial load was found in Gondar University teaching hospital. The higher bacterial load may be due to temperature, humidity, presence of unhygienic attached toilets, poor waste management system and poor ventilation system. Therefore, attention must be given to control those environmental factors which favor the growth and multiplication of microbes in indoor environment. In addition, also the ventilation condition, cleanliness of toilets, sweeping methods and waste disposal system of the compound should be improved.

## Background

Exposure to microorganisms suspended in the air of both occupational and residential indoor environments is associated with a wide range of adverse health effects with major public health impacts. The quality of indoor air is one of the most significant factors affecting the health and well being of people who inhale at least 10 m^3^ of the air every day, and spend between 80–95 % of their lives indoors [1, 2].

The air inhaled by people is abundantly populated with microorganisms which are also called bioaerosols. Bioaerosol is a colloidal suspension, formed by liquid droplets and particles of solid matter in the air, whose components contain or have attached to them viruses, fungal spores and conidia, bacterial endospores, plant pollen and fragments of plant tissues. They account for 5–34 % of indoor air pollution [3–5].

In many environments such as hospitals, the presence of bioaerosols can compromise normal activities. Infectious aerosols tend to be extremely small (<5 μm) and can, therefore, remain suspended and viable in the air stream over long periods of time, resulting in extremely high risk of airborne infection in confined places. Nosocomial infection is a serious and widespread problem with many of the infections associated with person to person contact with an estimated 1 in 10 patients acquiring an infection during a hospital stay. While many of these infections are associated with person-to-person contact, there is increasing evidence that some infections are transmitted by the airborne route. It has been calculated that the airborne route of transmission may account for as much as 10–20 % of all endemic nosocomial infections [2, 6].

Indoor air pollution is among the leading avoidable causes of disease and death. Globally, 4.3 million deaths were attributable to household air pollution in 2012, almost all in low and middle income countries. The South East Asian and Western Pacific regions bear most of the burden with 1.69 and 1.62 million deaths, respectively. Almost 600,000 deaths occur in Africa, 200,000 in the Eastern Mediterranean region, 99,000 in Europe and 81,000 in the Americas. The remaining 19,000 deaths occur in high income countries [7, 8]. The health burden from indoor air pollution can also be expressed in Disability Adjusted Life Years (DALYs). The WHO [9] reports that 41 million DALYs were lost due to indoor air pollution. Eleven percent of all deaths in low income countries are due to lower respiratory infections which are caused by indoor air pollution [10].

In hospitals, the problem of *Staphylococcus aureus* and *Streptococcus pyogenes* is a global public health problem, but it is particularly serious in resource limited countries. The most common skin bacterial infections are caused by *Staphylococcus aureus* and *Streptococcus pyogenes. Staphylococcus aureus* and *Streptococcus pyogenes* are general pathogens found in hospitals which may cause severe invasive infections [11].

For this study, we used settle plates technique to estimate bacterial load in the indoor air of wards. Passive air sampling uses “settle plates”, which are standard Petri dishes containing culture media, which are exposed to the air for a given time in order to collect biological particles which “sediment” out and are then incubated. According to some authors, passive sampling provides a valid risk assessment as it measures the harmful part of the airborne population which falls on to a critical surface, such as in the surgical cut or on the instruments in operating theatres [12]. In addition, active air sampling is applicable when the concentration of microorganisms is not very high. However, hence building and environmental conditions of the hospital are very poor; we suspect that there will be very high concentration of microorganisms [13–16].

## Methods

### Aims of the study

This study was conducted to assess bacteriological concentration of the indoor air of Gondar University teaching hospital. The study was also aimed to identify specific types of bacteria, namely *Staphylococcus aureus* from *Staphylococcus* species and *Streptococcus pyogenes* from *Streptococcus* species which have high public health significance.

### Study design

Cross-sectional study was conducted to assess the bacteriological concentration and to identify specific species of bacteria in the indoor air of Gondar University teaching hospital.

### Sampling procedures

Air samples were taken from 14 randomly selected wards of the hospital, namely surgery, emergency, orthopedic, general ward, radiology, obstetric, medical ward, psychiatry, optometry, TB ward, ward C, ward D, physiotherapy, and kalaazar wards which provided patient care services at the time of data collection.

Bacterial measurements were made by passive air sampling technique i.e., the settle plate method using 9 cm diameter Petri dishes. In each ward five Petri dishes were exposed for 30 and 60 min in the morning and afternoon. The sampling height which approximated to human breathing zone was 1 m above the floor and at the center of the room. To minimize dilution of air contaminants, openings like doors and windows were closed including the mechanical ventilators during sampling. In addition, the movement of people during sampling was restricted to avoid air disturbance and newly emitted microorganisms. Bacteria were collected on nutrient agar and blood agar media. To obtain the appropriate surface density for counting and to determine the load with respect to time of exposure, the sampling times were set at 30 and 60 min in the morning (at 8:00 AM) and afternoon (2:00 PM).

By this survey building related factors (like sweeping methods of the floor, design, presence of attached toilets, ventilation, temperature and humidity) and compound sanitation (i.e., waste management systems) were assessed.

### Air sample analysis

Both quantitative and qualitative analyses were conducted. The quantitative analysis was mainly conducted to determine bacterial load or number of bacteria in the indoor air. To determine the load, exposed culture medias/ air samples were taken to the laboratory and incubated at 37 °C for 24 h. After 24 h incubation period, bacterial load was enumerated as colony forming units (CFU) and CFU/m^3^ were determined by the formula ***N*** = 5a*10^**4**^**(bt)**^**−1**^ [17–19], where *N* = microbial CFU/m^3^ of indoor air; a = number of colonies per Petri dish; b = dish surface (cm^2^); and t = exposure time (minutes). Besides, one way ANOVA was also conducted to obtain the mean bacterial concentration of wards.

Qualitative analysis was conducted to identify specific species of bacteria. For this study we have selected *Staphylococcus aureus and Streptococcus* which had high public health concern. Mannitol test was used to isolate *Staphylococcus aureus*, and Bacitracin test was conducted to isolate *Streptococcus pyogenes*.

*Staphylococcus aureus* was isolated by Mannitol Salt Agar Plate, a selective agar medium which inhibits the growth of most bacteria other than staphylococcus species especially *Staphylococcus aureus*.

Bacitracin test is sensitivity test used to differentiate the beta- hemolytic *Streptococcus. Streptococcus pyogenes* (group A streptococci) is bacitracin sensitive species and inhibited by the small amount of bacitracin in the disk. Any zone of inhibition around the disk indicates positive result and no zone of inhibition indicates negative result.

### Sample quality

To secure the quality of the study, aseptic techniques like utilization of safety clothes; sterilization of sampling utensils; cold storage and handling of serialized utensils; proper incubation of samples were applied. Field blanks were also used to check the presence of cross contamination.

## Results

### Bacterial load

**Fig. 1:**
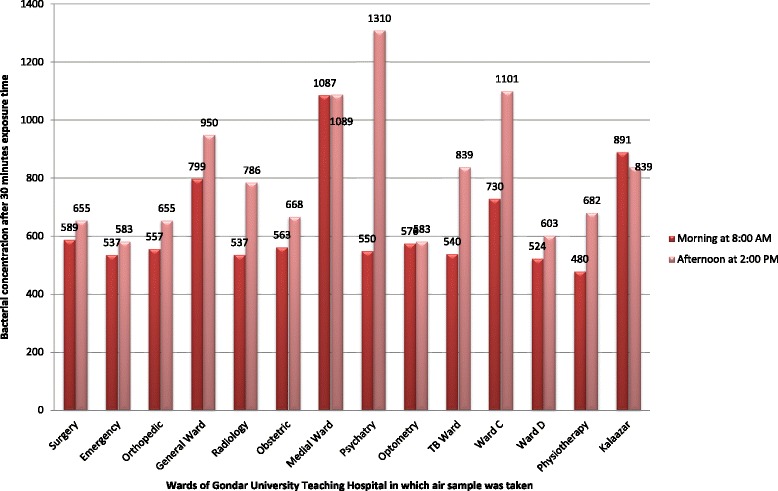
Bacteriological concentration of indoor air of Gondar University teaching hospital after 30 min exposure time, Gondar town, Northwest Ethiopia, 2015

**Fig. 2:**
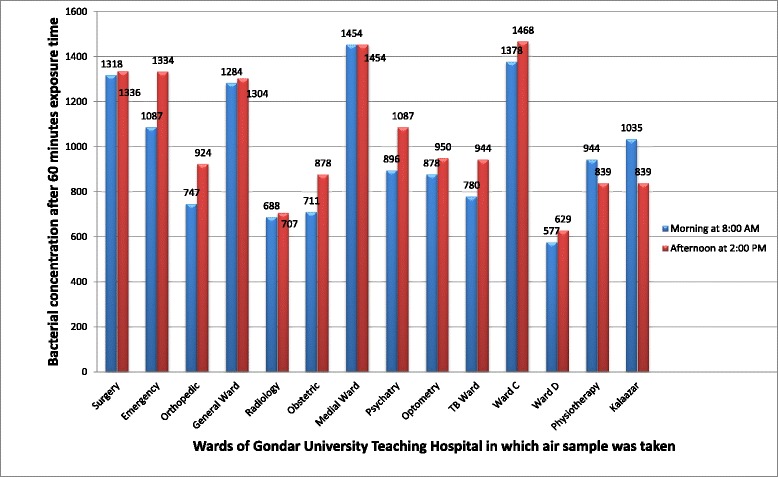
Bacteriological concentration of indoor air of Gondar University teaching hospital after 60 min exposure time, Gondar town, Northwest Ethiopia, 2015

The result of this study indicated that the highest bacterial load (which was 1468 CFU/m^3^) has been found at 2:00 PM in Ward C at 60 min exposure time and the lowest bacterial concentration (i.e., 480 CFU/m^3^) was recorded at 8:00 AM in physiotherapy ward at 30 min exposure time (Figs. 1 and 2).

### Specific types of bacteria

In this study two species of bacteria which have high public health significance, namely *Staphylococcus aureus* and *Streptococcus pyogenes* were isolated. As the test result indicated, these two bacteria are found in most of the wards (Table 1).

**Table 1:** Bacteria isolated from each investigated ward of Gondar University teaching hospital, Gondar town, Northwest Ethiopia, 2015

Investigated wards	Staphylococcus aureus	Streptococcus pyogenes
Surgery	+	+
Emergency	+	-
Orthopedic	_	+
General ward	+	+
Radiology	+	_
Obstetric	+	_
Medical ward	+	_
Psychiatry	_	_
Optometry	+	_
TB ward	+	+
Ward C	+	+
Ward D	+	+
Physiotherapy	_	+
Kalazar ward	_	+

### Building and compound condition

The result of this survey revealed that wards are constructed in areas prone to contamination. The site was characterized by indiscriminate waste disposal. Unhygienic attached toilets were observed. Microorganisms from these unhygienic toilets may transmit to the wards by people or air current. There were no mechanical ventilations in any of the wards. As a result, buildings are ventilated by the aid of natural ventilation system which may increase the possibility of entrance of pollutants from the unhygienic external environment. Even though wet moping was recommended to reduce aerosols, dry sweeping was being practiced which may increase bio – aerosols in the indoor air. The temperature (26.5–29.5 °C) and humidity (64.5–85 %) of each ward were at the favorable range for the growth and multiplication of bacteria.

### ANOVA test for mean bacterial concentration

One way ANOVA test was conducted to obtain the mean bacterial concentration of wards as presented below. The highest mean bacterial concentration (1271.00 CFU/m^3^) was found in Medical ward and the least (583.25 CFU/m^3^) concentration was found in Ward D. The grand total average concentration was 878.43 CFU/m^3^ (Table 2).

**Table 2:** One way ANOVA results for mean bacterial concentration of wards in Gondar University Teaching Hospital, 2015

Wards	Mean	Std. error	95 % Confidence interval for mean		Minimum	Maximum
			Lower bound	Upper bound		
Surgery	974.5000	203.99449	325.2985	1623.7015	589.00	1336.00
Emergency	885.2500	194.66055	265.7533	1504.7467	537.00	1334.00
Orthopedic	720.7500	78.06875	472.3004	969.1996	557.00	924.00
General ward	1084.2500	125.02691	686.3586	1482.1414	799.00	1304.00
Radiology	679.5000	52.02323	513.9389	845.0611	537.00	786.00
Obsetetric	705.0000	65.50954	496.5194	913.4806	563.00	878.00
Medical ward	1271.0000	105.65589	934.7558	1607.2442	1087.00	1454.00
Psychatry	960.7500	160.94066	448.5650	1472.9350	550.00	1310.00
Opthometry	746.7500	97.68433	435.8749	1057.6251	576.00	950.00
TB ward	775.7500	85.58853	503.3691	1048.1309	540.00	944.00
Ward c	1169.2500	165.93843	641.1599	1697.3401	730.00	1468.00
Ward d	583.2500	22.42162	511.8944	654.6056	524.00	629.00
Physiotherapy	736.2500	100.96400	414.9375	1057.5625	480.00	944.00
Kalazar	1005.7500	93.74556	707.4098	1304.0902	839.00	1258.00
Total	878.4286	38.99062	800.2896	956.5675	480.00	1468.00

ANOVA test result was presented to show the mean bacterial concentration difference among different wards. The test showed that there was significant mean bacterial concentration difference among wards (Table 3).

**Table 3:** ANOVA test result on mean bacterial concentration difference among different wards

Source of variation	Sum of squares	df	Mean square	F	Sig.
Between groups	2172222.214	13	167094.016	2.796	0.006
Within groups	2510205.500	42	59766.798		
Total	4682427.714	55			

## Discussion

In this study the bacterial concentration of indoor air of Gondar University teaching hospital wards was found in the range between 480 and 1468 CFU/m^3^. This range of bacterial load is much lesser than that reported from Jima University specialized hospital in which it was estimated between 2123 and 9733 CFU/m^3^ [20]. Though there is no uniform international standard available on levels and acceptable maximum bacterial loads in indoor air, the work conducted by a WHO expert group on assessment of health risks of biological agents in indoor environments suggested that total microbial load should not exceed 1000 CFU/m^3^ [21], whereas other scholars considered that 750 CFU/m^3^ should be the limit for bacteria [22, 23]. Airborne microbial concentrations ranging from 4500 to 10,000 CFU/m^3^ also have been suggested as the upper limit for ubiquitous bacterial aerosols [24]. The sanitary standards of European Commission for non industrial premises consider less than 50 CFU/m^3^ as ‘very low’ bacterial load, 50–100 CFU/m^3^ as ‘low’, 100–500 CFU/m^3^ as ‘intermediate’, 500–2000 CFU/m^3^ as ‘high’ and above 2000 CFU/m^3^ as ‘very high’ load [25]. According to these standards the bacterial load of Gondar University teaching hospital is considered as ‘high’.

This study revealed that there were no mechanical ventilations in any of the wards. Buildings were being ventilated by the aid of natural ventilation system which may increase the possibility of entrance of pollutants from the unhygienic external environment. This might be the reason why bacterial load was higher in different wards as a number of other studies also indicated that insufficient ventilation system contributes to the high microbial loads of the wards [26–30].

In this study, temperature and humidity of the wards also were measured. The temperature (26.5–29.5 °C) and humidity (64.5–85 %) ranges of the wards recorded are generally favorable for survival and multiplication of microorganisms. Temperature and humidity range unfavorable for microbial growth is between 20 and 22 ° C and 30–60 %, respectively. This might be the reason for high load of microorganisms in different wards of Gondar University teaching hospital as also suggested by other similar studies [27, 29, 31, 32].

Presence of unhygienic attached toilets and poor waste management system were observed during the survey. These conditions do have certain implications with regard to indoor air quality. Toilets and waste disposal sites should be located at a significant distance away from the hospital wards as they could be a potential source of infection. This study suggests that the higher bacterial load in the wards may be due to the presence of unhygienic attached toilets in the wards and poor waste management system. This explanation is supported by the results of other researches as well [33–35].

## Conclusions

Compared with different indoor air biological standards, higher bacterial concentration of indoor air was found in Gondar University teaching hospital. The higher bacterial load may be due to temperature, humidity, insufficient ventilation, presence of unhygienic attached toilets and poor waste management system. Hence the bacterial load is very high in all wards; attention should be taken for the immunocompromised patients. Attention must be also given to control those environmental factors which favor the growth and multiplication of microbes in indoor environment. In addition, also the ventilation condition of the wards, cleanliness of toilets and waste disposal system should be improved.

## Ethics approval

Ethical clearance was obtained from Institutional Ethical Committee of University of Gondar. Then, official letter from the University of Gondar Research and Community Service Vice President and supportive letter from college of Medicine and Health sciences was written to the respective responsible bodies. Confidentiality of the data was maintained. No identifiers except coding were included in the data collection tools.

## Abbreviations

μm: micrometer
AM: ante meridian
ANOVA: analysis of variance
CFU: colony forming units
cm^2^: centimeter square
DALYs: disability adjusted life years
m^3^: cubic meter
°C: degree centigrade
PM: post meridian
WHO: World Health Organization
